# Empowering patients through effective communication: The teach-back method as a tool for health literacy

**DOI:** 10.34172/hpp.025.44214

**Published:** 2025-11-04

**Authors:** Hassan Mahmoodi

**Affiliations:** ^1^Department of Public Health, Faculty of Health, Kurdistan University of Medical Sciences, Sanandaj, Iran; ^2^Social Determinants of Health Research Center, Research Institute for Health Development, Kurdistan University of Medical Sciences, Sanandaj, Iran

## Introduction

 Effective health communication is paramount for ensuring patients understand their health conditions, treatment plans, and self-care instructions.^1^ However, numerous barriers, such as medical jargon, complex information, and low health literacy, can hinder effective communication and lead to poor patient outcomes.^2^ To bridge this gap, healthcare professionals must cultivate strong communication skills, and the teach-back method emerges as a crucial strategy.^3^

 The teach-back method is a patient-centered communication technique in which healthcare providers convey important medical information—such as diagnoses, treatment plans, or self-care instructions—and then ask patients to restate the information in their own words. This process helps confirm the patient’s understanding, identify any misunderstandings, and allows the provider to re-explain the content if needed. Rather than testing the patient, the method is designed as a respectful way to ensure that healthcare information is delivered clearly and is truly understood.^4^ This method is not a test of the patient, but rather a test of whether or not the information has been understood well. It is especially useful in improving health literacy, reducing medical errors, and enhancing patient outcomes.

 This simple yet powerful technique allows providers to assess patient comprehension, identify knowledge gaps, and clarify any misconceptions. By actively involving patients in the learning process, the teach-back method empowers them to become active participants in their own healthcare.^5,6^

 While prior studies, including Yen and Leasure, have demonstrated the effectiveness of the teach-back method in improving patient education and outcomes, our letter focuses on practical implementation strategies and advocates for the systematic integration of the method into healthcare training programs.^3^ Unlike previous reviews that primarily assessed effectiveness, we aim to bridge the gap between knowledge and action by providing specific recommendations for adoption.

 A systematic review found that the teach-back method was effective in improving health literacy among patients with chronic diseases, demonstrating its superiority over traditional educational methods in enhancing knowledge retention and self-care behaviors.^7^ Teach-back is a valuable strategy that can improve the safety and quality of health care and supports the National Action Plan to Improve Health Literacy.^8^

 Despite growing evidence supporting the effectiveness of the teach-back method, there remains a gap in the literature regarding practical strategies for its widespread implementation in real-world clinical settings.^9^ Most prior studies have focused on outcome measurement rather than offering concrete guidance for adoption.^10^ This letter addresses that gap by presenting actionable recommendations for integrating the method into health professional training and daily practice. By translating evidence into practice, this approach has the potential to improve patient comprehension, reduce preventable errors, and ultimately enhance health literacy across populations—especially among individuals with limited literacy or chronic conditions who are most vulnerable to miscommunication.

## Practical implementation of the teach-back method

 Implementing the teach-back method effectively requires specific training for healthcare professionals. This training should encompass several key components, including:

- Understanding the core principles: Healthcare providers must grasp the rationale behind the method and its importance in promoting patient understanding.
- Developing strong communication skills: This includes using plain language, active listening, and non-judgmental questioning techniques to create a comfortable and supportive learning environment.
- Practicing the teach-back method: Role-playing and simulation exercises can provide valuable opportunities for healthcare providers to practice the teach-back method in a safe and controlled setting.
- Receiving feedback and ongoing support: Regular feedback and opportunities for skill refinement are essential to ensure continuous improvement in the use of the teach-back method.

By incorporating the teach-back method into their clinical practice, healthcare professionals can significantly enhance patient health literacy. Studies have shown that the teach-back method can improve patient adherence to treatment plans, reduce medication errors, and enhance overall patient satisfaction.^3,7^ Moreover, it fosters a stronger patient-provider relationship built on trust and mutual understanding. By prioritizing effective communication and utilizing patient-centered approaches like the teach-back method, healthcare providers can empower patients to make informed decisions about their health and improve their overall health outcomes.^11^

 In conclusion, strong health communication skills are essential for all healthcare professionals. By emphasizing the teach-back method, healthcare providers can effectively assess patient understanding, identify and address knowledge gaps, and ultimately improve patient health literacy. Investing in training programs that equip healthcare professionals with the necessary communication skills and the ability to utilize the teach-back method effectively is crucial for improving the quality of patient care and ensuring that patients have the information and support they need to make informed decisions about their health. I urge healthcare organizations to prioritize training initiatives that incorporate this method into clinical practice, as it holds the potential to empower patients and improve overall health outcomes.

## Competing Interests

 The author declares that he has no competing interests.

## Ethical Approval

 Not applicable.
